# Self-Assembled Polyelectrolyte Nanoparticles as Fluorophore-Free Contrast Agents for Multicolor Optical Imaging

**DOI:** 10.3390/molecules20034369

**Published:** 2015-03-09

**Authors:** Da Hye Shin, Min Beom Heo, Yong Taik Lim

**Affiliations:** 1 Center for Chemical analysis, Korea Research Institute of Chemical Technology (KRICT), Daejeon 305-600, Korea; E-Mail: sdh2080@krict.re.kr; 2 SKKU Advanced Institute of Nanotechnology (SAINT), School of Chemical Engineering, Sungkyunkwan University, Suwon 440-746, Korea; E-Mail: hmbgo@nate.com

**Keywords:** nanoparticles, optical imaging, self-assembly, cell labeling

## Abstract

In this work, we describe the fabrication of self-assembled polyelectrolyte nanoparticles that provide a multicolor optical imaging modality. Poly(γ-glutamic acid)(γ-PGA) formed self-assembled nanoparticles through electrostatic interactions with two different cationic polymers: poly(L-lysine)(PLL) and chitosan. The self-assembled γ-PGA/PLL and γ-PGA/chitosan nanoparticles were crosslinked by glutaraldehyde. Crosslinking of the ionic self-assembled nanoparticles with glutaraldehyde not only stabilized the nanoparticles but also generated a strong autofluorescence signal. Fluorescent Schiff base bonds (C=N) and double bonds (C=C) were generated simultaneously by crosslinking of the amine moiety of the cationic polyelectrolytes with monomeric glutaraldehyde or with polymeric glutaraldehyde. The unique optical properties of the nanoparticles that resulted from the crosslinking by glutaraldehyde were analyzed using UV/Vis and fluorescence spectroscopy. We observed that the fluorescence intensity of the nanoparticles could be regulated by adjusting the crosslinker concentration and the reaction time. The nanoparticles also exhibited high performance in the labeling and monitoring of therapeutic immune cells (macrophages and dendritic cells). These self-assembled nanoparticles are expected to be a promising multicolor optical imaging contrast agent for the labeling, detection, and monitoring of cells.

## 1. Introduction

The design and synthesis of functional nanomaterials have attracted considerable interest in the fields of biology and medicine because of their potential applications in the diagnosis and treatment of diseases [1,2,3,4,5]. Metal and semiconductor nanoparticles have been widely used as contrast agents for optical imaging due to their quantum effects [6,7,8,9]. Compared with conventional organic fluorophores, these inorganic nanoparticles have several advantages for use as optical imaging probes, such as high quantum yields, low photo-bleaching, multiplexed detection capability, and unique surface plasmon properties [10,11]. However, the potential toxicities of non-degradable inorganic nanoparticles limit their use in actual clinical applications.

Among the various biomedical imaging technologies, fluorescence optical imaging techniques have been widely used in biological and medical fields due to their high sensitivity and inexpensive instruments [12,13]. The ideal fluorophore for fluorescence imaging must function without being conjugated to any fluorescent materials and should be non-toxic because the fluorophore itself can alter the intrinsic behavior of labeling targets [14]. Based on these requirements, metal and semiconductor quantum dots are not ideal fluorescence imaging probes. Moreover, nanoparticles have some disadvantages, such as complex or expensive manufacturing processes and toxicity, despite their many advantages in terms of their photonic properties [1,2,8,15,16]. Recently, multifunctional nanoparticles that contained both fluorescence imaging probes and therapeutic drugs have been developed for theranostic applications [1,2,8,15,16]. In this process, various organic solvents were used to encapsulate fluorescence optical imaging probes into the nanoparticle matrix. Therefore, the development of novel type of optical fluorophores which have low toxicity and can be fabricated by organic solvent-free process is highly required.

In this study, we describe fluorophore-free autofluorescent nanoparticles as a novel type of optical imaging nanoprobe [17,18,19]. These nanoparticles are generated through the electrostatic assembly and crosslinking of biocompatible polyelectrolytes in aqueous solutions. Crosslinking of the ionic self-assembled polyelectrolyte nanogels with glutaraldehyde not only stabilized the nanogels but also generated a strong autofluorescence signal. We have also shown that this novel optical imaging nanoprobe exhibited high performance in the labeling and monitoring of therapeutic immune cells both *in vitro* and *in vivo*.

## 2. Results and Discussion

### 2.1. Fabrication and Characterization of Polyelectrolyte Nanoparticles

**Figure 1:**
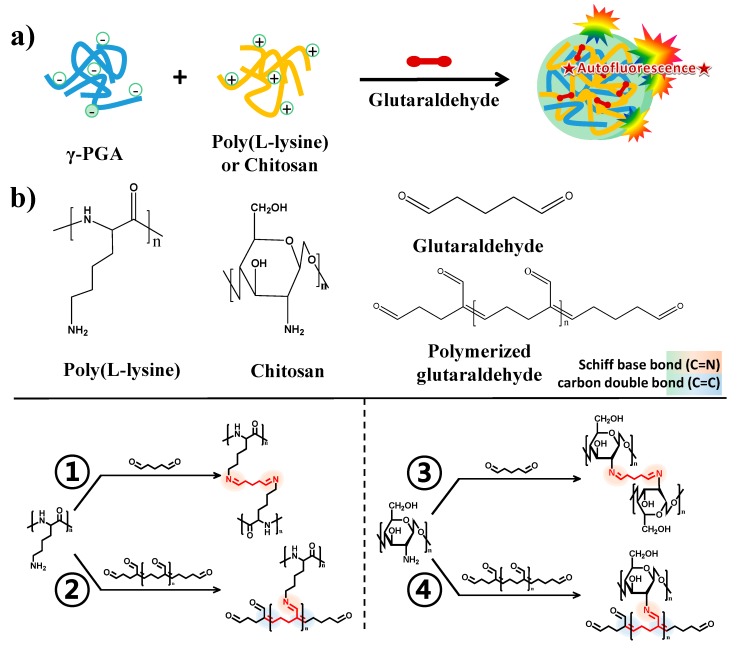

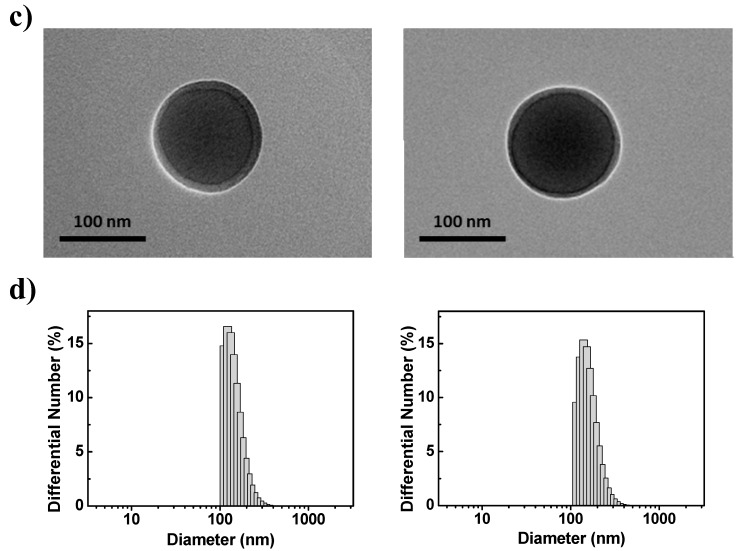
(**a**) Schematic illustration for the fabrication of autofluorescent nanocomposites based on electrostatic assembly and cross-linking by glutaraldehyde; (**b**) Chemical structure of cross-linked amino groups by glutaraldehyde monomer (PLL: ①, Chitosan: ③) and polymerized glutaraldehyde (PLL: ②, Chitosan: ④); (**c**) TEM images of γ-PGA/PLL (left) and γ-PGA/Chitosan (right) nanocomposites, respectively; (**d**) DLS analysis of γ-PGA/PLL (left) and γ-PGA/Chitosan (right) nanocomposites, respectively.

The synthesis of functional polyelectrolyte nanoparticles that provided optical imaging modalities was performed via electrostatic assembly and crosslinking without any fluorescent materials. The self-assembled polyelectrolyte nanoparticles were prepared via electrostatic interactions between the negatively charged carboxyl groups of poly(γ-glutamic acid) (γ-PGA) and the positively charged amino groups of two different polymers: poly-L-lysine (PLL) or chitosan. The crosslinking of amine moieties by glutaraldehyde in the self-assembled nanoparticles imparted higher stability and optical properties to the nanoparticles (Figure 1a). Crosslinking of the amine moiety of cationic polymers with glutaraldehyde provided structural integrity to the nanogels and involved the formation of a autofluorescent chemical bond. Fluorescent Schiff base bonds (C=N) and double bonds (C=C) could be generated simultaneously by crosslinking the cationic polymer with monomeric glutaraldehyde or by polymerizing glutaraldehyde into oligomeric glutaraldehyde followed by a reaction with the cationic polymer, respectively. The chemical structures that result from the Schiff base bonds (C=N) and carbon double bonds (C=C) of the polymerized glutaraldehyde provide autofluorescence properties for biological applications (Figure 1b). As shown in the transmission electron microscopy (TEM) images, the two types of synthesized nanoparticles exhibited a spherical shape (Figure 1c). The mean size of the nanoparticles was approximately 151.3 ± 39.0 nm in the case of γ-PGA/PLL and 167.3 ± 44.6 nm in the case of γ-PGA/chitosan (Figure 1d). The γ-PGA/PLL and γ-PGA/chitosan nanoparticles both possessed strong positive zeta potentials (+34.48 mV and +31.26 mV, respectively) in deionized water (DW).

### 2.2. Optical Properties of Self-Assembled Polyelectrolyte Nanoparticles

**Figure 2:**
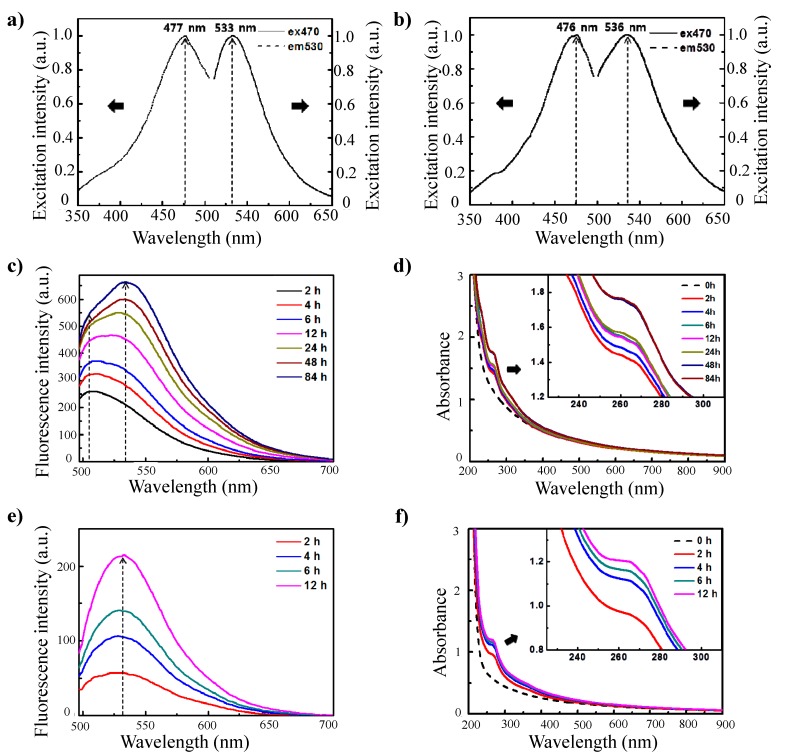
(**a**) Fluorescence excitation (λem: 530 nm) and emission (λex: 470 nm) curves of γ-PGA/PLL cross-linked with glutaraldehyde for 12 h; (**b**) Fluorescence excitation (λem: 530 nm) and emission (λex: 470 nm) curves of γ-PGA/Chitosan cross-linked with glutaraldehyde for 96 h; (**c**) Fluorescence emission spectra and (**d**) UV-vis spectra of γ-PGA/Chitosan cross-linked with 10 μL glutaraldehyde for different times (λex: 470 nm). (**e**) Fluorescence emission spectra and (**f**) UV-vis spectra of γ-PGA/PLL cross-linked with 10 μL glutaraldehyde for different times (λex: 470 nm).

These nanoparticles exhibited unique optical properties. The nanoparticles generated a strong fluorescence signal, and the shapes of the excitation and emission curves of these nanoparticles were similar to those of conventional fluorophores. The fluorescence spectrum of the γ-PGA/PLL nanoparticles revealed that emission occurs at 533 nm upon excitation at 470 nm (Figure 2a). When the γ-PGA/chitosan nanoparticles were excited at 470 nm, the emission peak was observed at 536 nm (Figure 2b). The maximum peak in the emission spectrum was highly dependent on the crosslinking reaction time, and the behavior of this peak was considerably different in the two nanoparticle systems. The required crosslinking reaction time to exhibit distinct emission was longer for the γ-PGA/chitosan nanoparticles than for the γ-PGA/PLL nanoparticles. The different dependencies of the emission spectra on the crosslinking reaction time in the two nanoparticle systems may be a result of the different chemical structures of the cationic polymers. As shown in Figure 1b, conjugation of the imine bond (C=N) and carbon double bond (C=C) causes an apparent fluorescence curve for their optical properties.

The fluorescence intensity of the γ-PGA/chitosan nanoparticles at λ_em_ = 533 nm improved with an increase in the crosslinking reaction time (Figure 2c). For the γ-PGA/chitosan nanoparticles, the primary emission occurred at λ_em_ = 510 nm for crosslinking reaction times up to 6 h. For reaction times longer than 6 h, peaks at λ_em_ = 510 nm and λ_em_ = 531 nm were observed with approximately equal intensities. This result suggested that the imine bonds coexist equally with carbon double bonds. The emission peaks measured after 12 h were clearly dominant at 531–534 nm. From this result, we could infer that Schiff base bonds are gradually formed with an increase in the reaction time and that the effects of C=N bonds and C=C bonds on the improvement in fluorescence intensity are different depending on the crosslinking reaction time. Furthermore, the formation of Schiff base bonds (C=N) was confirmed by measuring the absorbance of the particles (Figure 2) [20]. The gradual increase in absorbance by the γ-PGA/chitosan nanoparticles within the range of 250–270 nm indicated that the increase in fluorescence intensity was caused by the formation of imine bonds. This result, *i.e.*, the steady increase in fluorescence intensity was caused by the formation of imine bonds, was consistent with the spectra obtained from the γ-PGA/PLL nanoparticles (Figure 2e,f).

**Figure 3:**
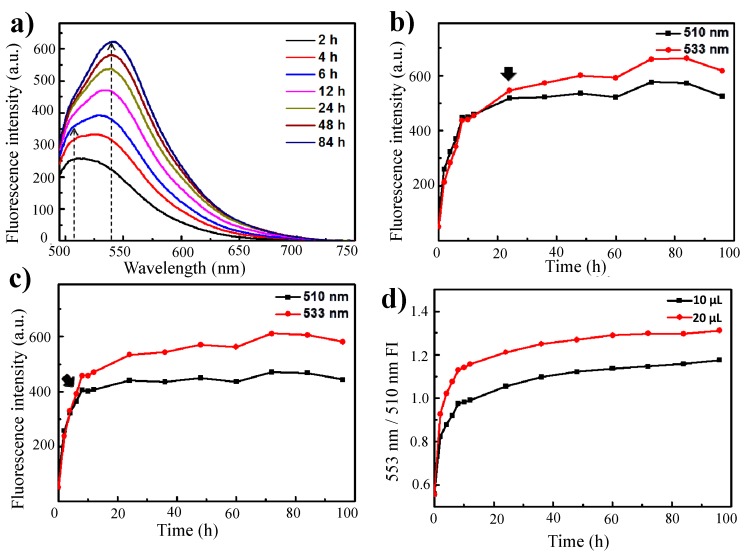
(**a**) Fluorescence emission spectra of γ-PGA/Chitosan cross-linked with 20 μL glutaraldehyde for different times (λex: 470 nm); The fluorescence intensity of (**b**) γ-PGA/Chitosan cross-linked with 10 μL glutaraldehyde and (**c**) γ-PGA/Chitosan cross-linked with 20 μL glutaraldehyde at 510 nm and 533 nm as a function of time; (**d**) Relationship between rates of formation of Schiff base, showed by calculating the ratio between fluorescence intensity at 533 nm and 510 nm.

Notably, the shapes of the fluorescence emission curves were dependent on the crosslinker volume and on the crosslinking reaction time. When more than 10 μL of glutaraldehyde was added to the γ-PGA/chitosan reaction mixture, the emission peaks were dominant within 6 h at 533 nm (Figure 3a). For γ-PGA/chitosan crosslinked with 10 μL of glutaraldehyde, the emission peak at 533 nm was more intense than the peak at 510 nm after 24 h of reaction, whereas the main emission peak of γ-PGA/chitosan crosslinked with 20 μL of glutaraldehyde decreased in intensity within 6 h (Figure 3b,c). As indicated by the ratios between the fluorescence intensity at 533 nm and that at 510 nm for γ-PGA/chitosan crosslinked with 10 μL and 20 μL of glutaraldehyde, the formation of Schiff base bonds was faster in γ-PGA/chitosan crosslinked with more glutaraldehyde (Figure 3d).

**Figure 4:**
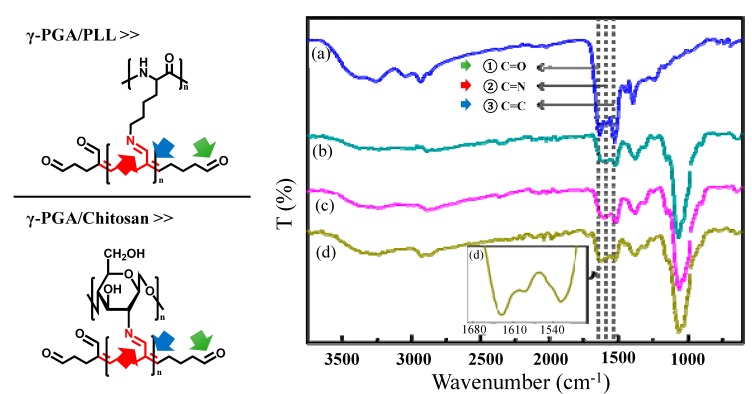
FT-IR spectra of (a) γ-PGA/PLL cross-linked with glutaraldehyde for 24 h, γ-PGA/Chitosan cross-linked with glutaraldehyde for (b) 30 min, (c) 24 h, and (d) 96 h. (① C=O (1629–1640 cm^−1^), ② C=N (1589 cm^−1^), ③ C=C (1514–1530 cm^−1^)).

These results indicated that both the crosslinking reaction time and the crosslinker volume are important factors in the formation of chemical bonds that induce the final autofluorescence. In addition, the presence of C=N and C=C bonds was confirmed from FT-IR spectra (Figure 4). We could analyze the peaks in the FT-IR spectra that corresponded to chemical bonds that induce autofluorescence. The peak at 1629–1640 cm^−1^ was attributed to the aldehyde groups of glutaraldehyde (Figure 4①), and the peak at 1514–1530 cm^−1^ was assigned to C=C bonds conjugated with aldehyde groups of polymerized glutaraldehyde (Figure 4③).

**Figure 5:**
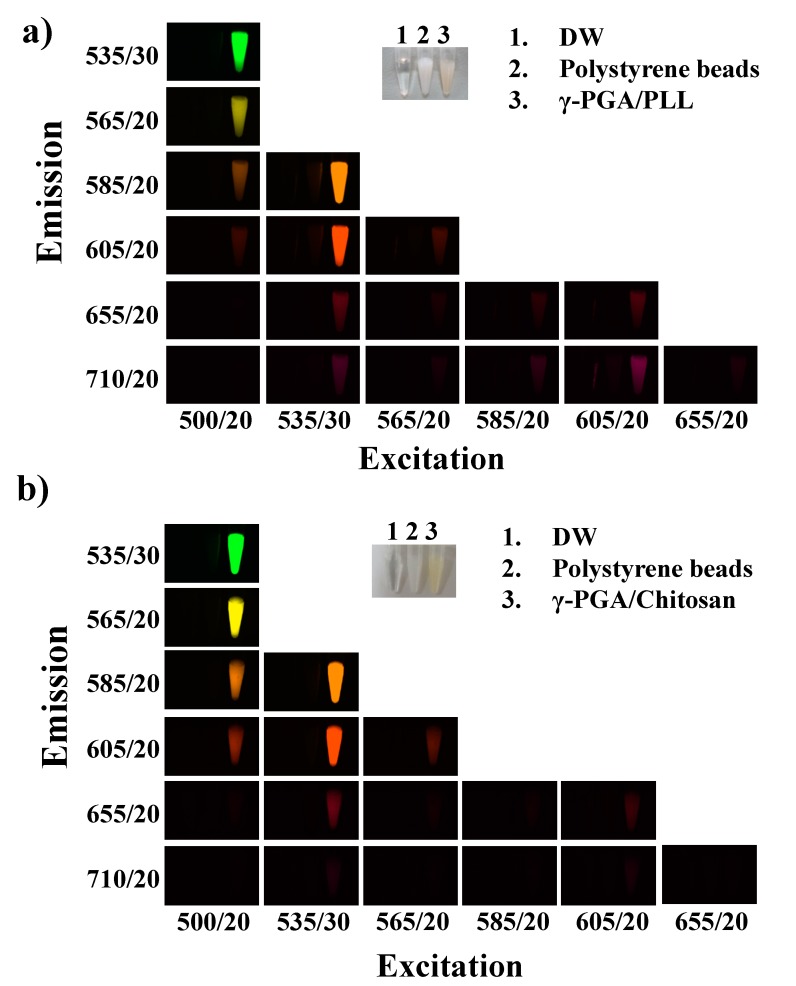
Fluorescence images of (**a**) γ-PGA/PLL cross-linked with glutaraldehyde for 12 h and (**b**) γ-PGA/Chitosan cross-linked with 10 μL glutaraldehyde for 96 h. (vertical axis: emission wavelength, horizontal axis: excitation wavelength, exposure time: 0.2 s).

The peak at 1589 cm^−1^ was attributed to the C=N bonds of the Schiff base (Figure 4②), which indicated that both γ-PGA/PLL and γ-PGA/chitosan nanoparticles exhibit autofluorescence as a result of the formation of these bonds. In the case of γ-PGA/chitosan nanoparticles, the gradual increase in the intensity of the peak at 1589 cm^−1^ was attributed to the formation of imine bonds with increasing crosslinking reaction time (Figure 4d). The principle of fluorescence is based on the transition of electrons between different orbitals. It is well-known that C=N bonds are associated with n-π* transitions and that C=C bonds are related to π-π* transitions [14]. Fluorescence images of the γ-PGA/PLL and γ-PGA/chitosan nanoparticles crosslinked with glutaraldehyde were obtained using suitable excitation and emission filters from the visible to near-infrared region (Figure 5). The γ-PGA/PLL and γ-PGA/chitosan nanoparticles both appeared to autofluorescence, without any fluorescent probes, depending on the optical filters used.

### 2.3. Cell Viability and Cellular Uptake

**Figure 6:**
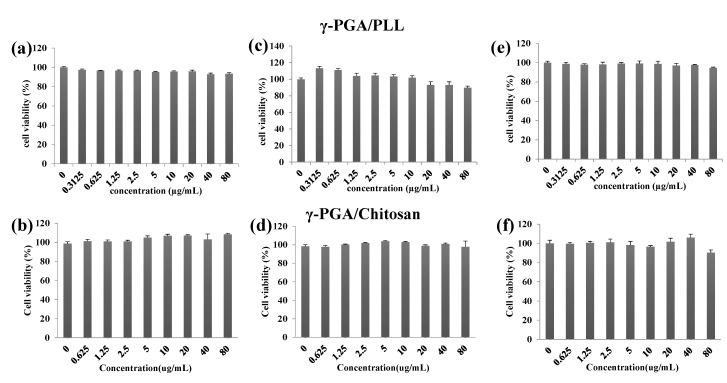
The cell viability of γ-PGA/PLL (up) and γ-PGA/Chitosan (down), determined by the MTS assay ((**a**,**b**) HeLa cells, (**c**,**d**) RAW264.7 cells, (**e**,**f**) DC2.4 cells). The two types of nanocomposites were incubated with the displayed concentrations (from 0.3125 µg/mL to 80 µg/mL) for 24 h. All experiments were performed in triplicate.

**Figure 7:**
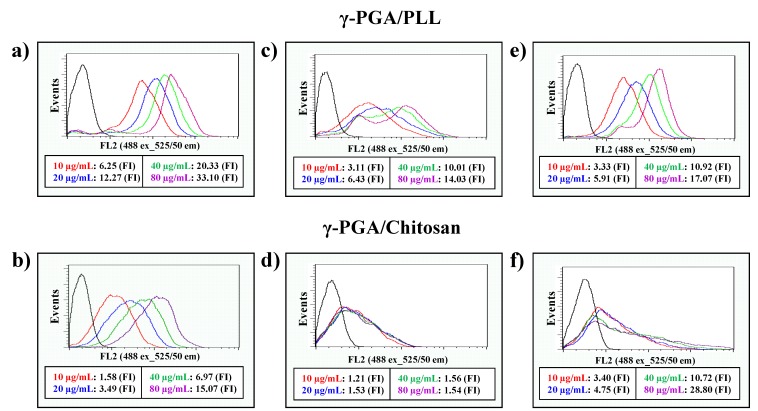
The flow cytometry analysis of γ-PGA/PLL (up) and γ-PGA/Chitosan (down), respectively ((**a**,**b**) HeLa cells, (**c**,**d**) RAW264.7 cells, (**e**,**f**) DC2.4 cells). The two types of nanocomposites were incubated with various concentrations (from 10 µg/mL to 80 µg/mL) for 24 h.

**Figure 8:**
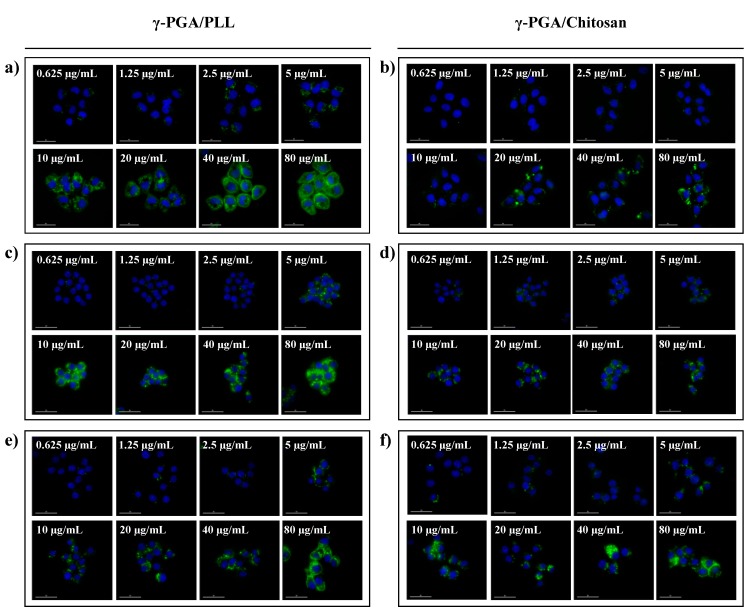
Fluorescence microscope images of γ-PGA/PLL (left) and γ-PGA/Chitosan (right), respectively ((**a**,**b**) HeLa cells, (**c**,**d**) RAW264.7 cells, (**e**,**f**) DC2.4 cells). The two types of nanocomposites were incubated with various concentrations (from 0.625 µg/mL to 80 µg/mL) for 24 h. (Ex 490/20; Em 526/36).

The cell viabilities of the γ-PGA/PLL and γ-PGA/chitosan nanoparticles were evaluated using an MTS assay *in vitro* (Figure 6). According to the results, the two types of nanoparticles were non-toxic to the cells. The intracellular delivery capacities of the two different nanoparticles were investigated using flow cytometry with various concentrations of the nanoparticles (Figure 7). The effects of the nanoparticles on cellular uptake were also investigated using fluorescence microscopy imaging (Figure 8). The intracellular uptake efficiency increased with increasing concentrations of nanoparticles. Regarding the charge effect on intracellular uptake, positively charged particles are more effective than negatively charged particles *in vitro* [21,22,23]. In the case of the γ-PGA/chitosan nanoparticles, the cellular uptake capacity was relatively lower because chitosan is deprotonated at neutral pH due to the pKa (pH 6.3) of the polymer [24]. As shown in Figure 8, the γ-PGA/PLL nanoparticles exhibited higher intracellular uptake efficiency than the γ-PGA/chitosan nanoparticles.

## 3. Experimental Section

### 3.1. Preparation of γ-PGA/PLL

γ-PGA/PLL nanoparticles were prepared via ionic self-assembly in aqueous solutions. The synthesis is based on the reaction between the carboxyl group of poly(γ-glutamic acid) (γ-PGA, 500 kDa, Bioleaders Corporation, Daejeon, Korea) and the amino group of poly-L-lysine (PLL, 15–30 kDa, Sigma-Aldrich, St. Louis, MO, USA). The detailed process for the synthesis of γ-PGA/PLL nanoparticles is as follows: γ-PGA (10 mg) in DW (1 mL) was added to PLL (18 mg) dissolved in DW (10 mL). The solution was stirred for 2 h at room temperature. For crosslinking of the nanoparticles, glutaraldehyde (10 μL, grade І, 50%, Sigma-Aldrich) was added as the crosslinking agent to the mixture and stirred for 1 h. To increase the stability of the nanoparticles, the mixture was PEGylated through the addition of methoxy poly(ethylene glycol)-succinimidyl glutarate (142 mg, mPEG-NHS, 5 kDa, SunBio, Inc., Anyang, Korea) for 12 h with stirring. To remove unreacted materials, such as mPEG-NHS, the PEGylated nanoparticles were washed three times with DW by centrifuging (6000 rpm) for 10 min each wash. Subsequently, the nanoparticles were redispersed in 4 mL of DW. During this step, a tip ultra sonicator (VCX750, Sonics &. Materials, Inc., Newtown, CT, USA) was applied for 10 s to completely disperse the nanoparticles.

### 3.2. Preparation of γ-PGA/Chitosan

The synthesis of the γ-PGA/chitosan nanoparticles is based on the reaction between the carboxyl group of poly(γ-glutamic acid) (γ-PGA, 2000 kDa, Bioleaders Corporation, Daejeon, Korea) and the amino group of chitosan (50 kDa, Youngchipharm Co., Ltd., Paju, Korea). After dissolving γ-PGA (20 mg) in DW (19 mL), the solution was acidified by adjusting the pH to 4.2. Next, chitosan (80 mg) in DW (1 mL) was added to the γ-PGA dissolved in DW, and then the mixture was stirred for 2 h. Glutaraldehyde (10 µL) was added to the solution. After reacting for 12 h for crosslinking, the nanoparticles were washed three times with DW by centrifuging (12,000 rpm) for 5 min each wash without the PEGylation process. Finally, the nanoparticles were dispersed in 4 mL of DW as described above.

### 3.3. Characterization of Nanoparticles

The sizes and size distributions of the nanoparticles were measured by dynamic light scattering (ELS-Z, Otsuka Electronics, Osaka, Japan). The surface of the nanoparticles was analyzed through zeta potential measurements using an ELS-Z. Images for characterizing the morphologies of the nanoparticles were obtained using a scanning electron microscope (SEM, JSM-7000F, JEOL Ltd. Tokyo, Japan) and a transmission electron microscope (TEM, JEM-2100F HR, JEOL Ltd.). The samples were prepared for the TEM and SEM observations by dropping the synthesized solution directly onto a 200-mesh copper grid coated with carbon film and a silicon wafer, respectively. Qualitative analysis of the imine bonds formed by the crosslinker was conducted using a Bruker Alpha-P FT-IR spectrometer (Bruker Optic GmbH, Ettlingen, Germany). Fluorescence spectra of the nanoparticles were obtained using a fluorescence spectrometer (LS 55 Luminescence Spectrometer, Perkin Elmer, Waltham, MA, USA), and UV/Vis spectra were recorded using a UV-Vis spectrophotometer (UV-1800, Shimadzu, Kyoto, Japan). Images for analyzing the fluorescence properties of the nanoparticles were obtained using a fluorescence imaging instrument developed in our laboratory.

### 3.4. Cell Viability Assay

HeLa, RAW264.7 and DC2.4 cells were incubated with various concentrations of γ-PGA/PLL and γ-PGA/chitosan nanoparticles for 24 h in flat-bottomed 96-well plates (Corning Costar, Cambridge, MA, USA) at a density of 1 × 10^4^ cells per well (100 µL). For the MTS assay, the Cell Titer 96 Aqueous One Solution kit (Promega, Madison, WI, USA) was used following the manufacturer’s protocols. Briefly, the MTS reagent was added (10 µL per well), and the plates were incubated for 3 h at 37 °C. The absorbance was detected at 490 nm with a microplate reader (VersaMax, Molecular Devices, Sunnyvale, CA, USA). All of the experiments were independently repeated three times.

### 3.5. Fluorescence Microscopy Imaging

To determine the intracellular delivery capacities of the γ-PGA/PLL and γ-PGA/chitosan nanoparticles, HeLa, RAW264.7 and DC2.4 cells were incubated with various concentrations of the nanoparticles (from 0.625 μg/mL to 80 μg/mL concentrations) in a µ-slide 8-well microscopy chamber at a density of 2 × 10^4^ cells per well for 24 h at 37 °C. The cells were then washed in cold PBS, fixed with 4% (w/v) paraformaldehyde solution for 20 min at room temperature and stained with 2 µg mL^−1^ Hoechst 33342 (trihydrochlor trihydrate, Invitrogen, Carlsbad, CA, USA) in PBS for 15 min. Fluorescence images were obtained using a DeltaVision PD (Applied Precision Technologies, Issaquah, WA, USA) with a filter set (excitation: 490/20, 555/25, and 645/30; emission: 526/36, 605/52, and 705/72) (Omega Optical, Brattleboro, VT, USA).

### 3.6. FACS Analysis

For the FACS analysis, HeLa, RAW264.7 and DC2.4 cells were seed on 6-well plates at a density of 5 × 10^5^ cells per well in culture medium. Various concentrations (from 0.625 µg/mL to 80 µg/mL) of γ-PGA/PLL and γ-PGA/chitosan nanoparticles were added to each well, and then the cells were incubated for 24 h. After washing with PBS, the cells were analyzed using MACS (Miltenyi Biotec, Bergisch Gladbach, Germany). A minimum of 10,000 events were collected. The data were analyzed using a MACSQuant Analyzer (Miltenyi Biotec).

## 4. Conclusions

In summary, we have developed two types of autofluorescent nanoparticles via the electrostatic self-assembly of polyelectrolytes and crosslinking with glutaraldehyde. The γ-PGA/PLL and γ-PGA/chitosan nanoparticles both exhibited strong fluorescence signals without any fluorescent materials. These interesting nanostructures exhibited outstanding improvements for optical imaging using fluorescence images through selection of appropriate wavelengths. It is clear that the two different nanoparticles enable us to obtain different visual contrasts in optical imaging for biomedical applications. Furthermore, the nanoparticles presented in this study have the potential to be used as therapeutic agents by encapsulating therapeutic drugs such as siRNA and anti-cancer drugs. Because of the multispectral capability of each particle, the nanoparticles presented in this study are anticipated to be a promising multimodal agent for labeling, detection, and monitoring in a variety of biological and biomedical applications.

## References

[1] Davis M.E. (2008). Nanoparticle therapeutics: An emerging treatment modality for cancer. Nat. Rev. Drug Discov..

[2] Nune S.K., Gunda P., Thallapally P.K., Lin Y.-Y., Laird Forrest M., Berkland C.J. (2009). Nanoparticles for biomedical imaging. Expert Opin. Drug Deliv..

[3] Ferrari M. (2005). Cancer nanotechnology: Opportunities and challenges. Nat. Rev. Cancer.

[4] Forrest M.L., Kwon G.S. (2008). Clinical developments in drug delivery nanotechnology. Adv. Drug Deliv. Rev..

[5] Weissleder R. (2006). Molecular imaging in cancer. Science.

[6] Salata O.V. (2004). Applications of nanoparticles in biology and medicine. J. Nanobiotechnol..

[7] Na H.B., Song I.C., Hyeon T. (2009). Inorganic nanoparticles for mri contrast agents. Adv. Mat..

[8] Sailor M.J., Park J.H. (2012). Hybrid nanoparticles for detection and treatment of cancer. Adv. Mat..

[9] Sajja H.K., East M.P., Mao H., Wang A.Y., Nie S., Yang L. (2009). Development of multifunctional nanoparticles for targeted drug delivery and non-invasive imaging of therapeutic effect. Curr. Drug Discov. Technol..

[10] Han M., Gao X., Su J.Z., Nie S. (2001). Quantum-dot-tagged microbeads for multiplexed optical coding of biomolecules. Nat. Biotechnol..

[11] Peer D., Karp J.M., Hong S., Farokhzad O.C., Margalit R., Langer R. (2007). Nanocarriers as an emerging platform for cancer therapy. Nat. Nanotechnol..

[12] Kim H.M., Noh Y.W., Park H.S., Cho M.Y., Hong K.S., Lee H., Shin D.H., Kang J., Sung M.H., Poo H. (2012). Self-fluorescence of chemically crosslinked mri nanoprobes to enable multimodal imaging of therapeutic cells. Small.

[13] Ntziachristos V. (2006). Fluorescence molecular imaging. Annu. Rev. Biomed. Eng..

[14] Wei W., Wang L.Y., Yuan L., Wei Q., Yang X.D., Su Z.G., Ma G.H. (2007). Preparation and application of novel microspheres possessing autofluorescent properties. Adv. Funct. Mater..

[15] Yu M.K., Park J., Jon S. (2012). Targeting strategies for multifunctional nanoparticles in cancer imaging and therapy. Theranostics.

[16] Prabhu S., Poulose E.K. (2012). Silver nanoparticles: Mechanism of antimicrobial action, synthesis, medical applications, and toxicity effects. Int. Nano Lett..

[17] Sharma P., Bengtsson N.E., Walter G.A., Sohn H.B., Zhou G., Iwakuma N., Zeng H., Grobmyer S.R., Scott E.W., Moudgil B.M. (2012). Gadolinium-doped silica nanoparticles encapsulating indocyanine green for near infrared and magnetic resonance imaging. Small.

[18] Hu J., Liu T., Zhang G., Jin F., Liu S. (2013). Synergistically enhance magnetic resonance/fluorescence imaging performance of responsive polymeric nanoparticles under mildly acidic biological milieu. Macromol. Rapid Commun..

[19] Shi B., Zhang H., Qiao S.Z., Bi J., Dai S. (2014). Intracellular microenvironment‐responsive label-free autofluorescent nanogels for traceable gene delivery. Adv. Healthc. Mater..

[20] Tong W., Gao C., Möhwald H. (2005). Manipulating the properties of polyelectrolyte microcapsules by glutaraldehyde cross-linking. Chem. Mater..

[21] Foged C., Brodin B., Frokjaer S., Sundblad A. (2005). Particle size and surface charge affect particle uptake by human dendritic cells in an in vitro model. Int. J. Pharm..

[22] Fröhlich E. (2012). The role of surface charge in cellular uptake and cytotoxicity of medical nanoparticles. Int. J. Nanomedicine.

[23] Nafee N., Schneider M., Schaefer U.F., Lehr C.-M. (2009). Relevance of the colloidal stability of chitosan/PLGA nanoparticles on their cytotoxicity profile. Int. J. Pharm..

[24] Helander I., Nurmiaho-Lassila E.-L., Ahvenainen R., Rhoades J., Roller S. (2001). Chitosan disrupts the barrier properties of the outer membrane of gram-negative bacteria. Int. J. Food Microbiol..

